# Burden and Correlates of HIV Risk among Men Who Have Sex with Men in Nagaland, India: Analysis of Sentinel Surveillance Data

**DOI:** 10.1371/journal.pone.0117385

**Published:** 2015-02-17

**Authors:** Malay Kumar Saha, Tanmay Mahapatra, Subrata Biswas, Piyali Ghosh, Medo Kire

**Affiliations:** 1 Virology Division, National Institute of Cholera and Enteric Diseases, Kolkata, West Bengal, India; 2 Epidemiology Division, National Institute of Cholera and Enteric Diseases, Kolkata, West Bengal, India; 3 Monitoring and Evaluation Division, Nagaland State AIDS Control Society, Kohima, Nagaland, India; University of Athens, Medical School, GREECE

## Abstract

**Background:**

Dynamics of HIV epidemic are largely understudied among men having sex with men (MSM) in India, while their potentially critical role in HIV spread is often stressed. Unfortunately, the epidemic has probably concentrated in this hard-to-reach population in the north-eastern high HIV-prevalent areas, especially in the bordering state of Nagaland, where HIV prevalence among MSM was found to be 2^nd^ highest in the whole country. Dearth of information regarding the socio-behavioral correlates of HIV acquisition among MSM in this remote hilly region thus called for detailed analyses of the HIV Sentinel Surveillance (HSS) data.

**Methods:**

During the first ever conducted HSS among MSM in Nagaland, between March and May, 2011, as per the operational guideline of Indian National AIDS Control Program, 243 MSM were recruited, interviewed and tested for HIV. Anonymous data on socio-demographics, sexual behavior and laboratory results were analyzed using SAS version-9.2 to conduct descriptive and logistic regression analyses.

**Results:**

Among the recruited MSM, mean age was 28.30 years, 46.09% were illiterate, 27.16% were unemployed, 57.02% identified them as Kothi (predominantly receptive anal sex partner), 14.81% were bisexual, 19.75% exchanged money for sex (within last 1 year) with men and 13.58% were HIV sero-positive. Increasing age (for 25–34yrs, adjusted odds ratio: AOR = 3.89, p = 0.046; reference = <25yrs), middle school (AOR = 3.44, p = 0.046) or higher (AOR = 4.47, p = 0.034) education (reference = illiterate), being Kothi [AOR = 3.60, p = 0.026; reference = double-decker: (involved in both insertive and receptive roles)] and having paid and received money for sex with a man (AOR = 7.32, p = 0.026; reference = didn’t exchange money) were strongly associated with higher risk of HIV in both bivariate and multivariate analyses.

**Conclusion:**

HIV burden was found to be alarmingly high among MSM in Nagaland. Targeted interventions for high-risk MSM, especially those who were older, educated, self-identified as kothis and involved in paid sex, seemed to be the need of the hour.

## Introduction

The estimated number of people living with HIV in India was 2.09 million in 2011 with an adult HIV prevalence of 0.27% [1]. Since the detection of the first case in 1986 among female sex workers (FSW), the HIV epidemic in this country has gone through many changes [1–4]. Although the predominant mode of transmission still remains to be heterosexual, the epidemic is now mainly concentrated among other high risk groups like transgender (TG), injecting drug users (IDU) and men who have sex with men (MSM), with national HIV prevalence of 8.82%, 7.14% and 4.43% respectively in 2011[1,4].

Social stigma, discrimination and abuse [5–7] have forced Indian MSM to be mostly hidden and marginalized [8–12]. Unlike the western world they do not have any distinct homosexual identity [8,10,12]. The most common self-perceived MSM categories identified here are Kothis (commonly the receptive partner), Panthis (usually insertive) and Double-deckers (engaged in both roles) [8,11–13]. As per the cultural pressure and socials norms, many of them get married and engaged in heterosexual activities [5,6,8,13,14], while their homosexual behaviors remain unknown to their family members [5,13]. The resultant diverse sexual mixing pattern, poor access to awareness programs and less utilization of the preventive measures expose MSM to high risk of acquiring and transmitting HIV [3,4,12]. Accordingly, the risk of HIV acquisition among the unassuming female partners of married MSM also increases, culminating into the possibility of upsurge in HIV epidemic in otherwise low-risk general population [12]. This potentially dangerous bridging role of the hard-to-reach MSM population remained understudied in most part of India, including Nagaland, one of the highest HIV prevalent state (having an adult HIV prevalence of 0.73% in 2011) in northeastern part of the country [4].

Nagaland is characterized by a highly diverse population with 16 tribes living in the state. With majority of population belonging to Christian population this state draws its culture from many other neighboring regions. As per the Census 2011, Nagaland has a population of 1.979 millions, with a gender ratio of 931 females per 1000 males and a population density of 119/km^sq^, much lower than Indian national average of 382/km^sq^. The literacy rate here is 79.55% (female literacy is 70.01%), human development index (HDI) is 0.64 and 18.88% of the population is below poverty line [15, 16].

Although the epidemic was initially concentrated among IDUs [2, 4] in this state, currently it is becoming more generalized and exhibiting a dual nature [2–4]. While HIV prevalence in Nagaland is declining among IDUs [2–4] it is the only high prevalent state where the trend is upward among FSWs [2,4] and this state currently has the highest HIV sero-positivity (0.84%) among pregnant women in the whole country [1]. As unprotected sex has now emerged as the commonest route of HIV transmission in Nagaland [4] and partner’s sexual behavior is considered to be the most important contributing factor for HIV acquisition among married women in India [17] initiatives to explain this dual HIV epidemic called for an evaluation of the role of MSM in the dynamics of HIV transmission in this state [2].

The prevalence of HIV and its risk factors among MSM remained unknown in Nagaland [2, 4] as HIV Sentinel Surveillance (HSS) in this mostly hidden population could start here only in 2011 [2]. To assess the adequacy of public health response for optimization of the targeted interventions aiming at harm reduction and prevention, some insight into the HIV situation among MSM in this state were urgently required [2]. Keeping this objective in mind, a cross-sectional analysis of HSS 2011 data was undertaken, to estimate the HIV burden and identify its socio-behavioral correlates in this population. To the best of our knowledge this was the first effort to measure the association between socio-behavioral factors and HIV burden among MSM in Nagaland.

## Materials and Methods

### Study Site

The HSS site for MSM in Nagaland is located in Dimapur, the largest city (0.38 million population) and the district with highest HIV prevalence (1.2% in 2007) in the state [2, 18].

### Study Design

A cross-sectional analysis of National HSS data collected during 2011 from designated sentinel site for MSM in Dimapur, Nagaland was conducted.

### Ethics Statement

All procedures of HIV sentinel surveillance (HSS) was conducted following Unlinked Anonymous Testing strategy approved by Ethical Committee of National AIDS Control Organization, New Delhi. The study involving human participants is in compliance with the Helsinki declaration. The participants gave written informed consent for participation. The written informed consent procedure for minor includes both assent by the participant and consent from the next to kin, caretakers, or guardian on behalf of the minors. The Institutional Ethics Committee, National Institute of Cholera and Enteric Diseases also approved the study.

### Inclusion Criteria

Men aged 15–49 years who had anal or oral sex with a male partner in last one month, visited the sentinel site during the HSS in 2011, not already approached for participating in the current round and agreed to participate were included in the study [19].

### HSS in Nagaland

HSS involving MSM population of the Nagaland state of India was conducted for the first time in the Dimapur district between the months of March to May, 2011. The sentinel site [which was a service point/clinic for sexually transmitted infections (STI)] was established by Guardian Angel, a non-Government organization (NGO) who started the Targeted Intervention project on MSM in this state with a local community based organization for MSM named Goodwill Ashram, under supervision of Nagaland State AIDS Control Society and National AIDS Control Organization (NACO) of India [20]. Information regarding the available services and surveillance procedures were conveyed to the target population through peer outreach workers and MSM networks by Guardian Angel. From the start of the surveillance period, each MSM who visited the sentinel site was enrolled consecutively after the assessment of the eligibility criteria [21]. After the collection of written informed consent the enrolled MSM were interviewed by the counselor to collect socio-behavioral information without any personal identification information. Blood samples from each subject were also collected by trained lab-technicians in the form of dried blood spot (DBS) following unlinked anonymous testing strategy as per the operational guidelines of NACO [4]. The data forms and DBS were sent periodically to respective Regional Institute for HSS and sample testing laboratory for further processing [21].

### Operational Guideline of Indian National AIDS Control Program

The HSS conducted in India by NACO, under Indian National AIDS Control Program, is the largest HSS system in the world. According to the operational guideline of the program using consecutive sampling strategy, unlinked anonymous testing for HIV is done for each recruited subjects for each high-risk groups (HRG). Blood samples collected in DBS are used for the diagnosis of HIV using a highly sensitive test followed by a highly specific test (for those who were positive in the first test) [19, 21].

### Data Collection

Altogether 243 eligible MSM who attended the sentinel site during this period were recruited and interviewed using a short (to make it less time-consuming to increase compliance) interviewer administered questionnaire. Anonymous information on socio-demographic and behavioral characteristics was collected without any personal identifiers. At the end of the interview, services (counseling/medical advice etc.) for which participants came to the site were provided. Quality of data collection and database management were ensured at each step by proper documentation and multiple logic checks.

### HIV Testing

Unlinked anonymous HIV testing of each participant was conducted using dried blood spot sample [19]. Samples were labeled with unique codes without any personal identifier to maintain anonymity and screened for HIV using sensitive ELISA (Microlisa HIV; J Mitra and Company Pvt Ltd, New Delhi, India). Positive samples were then retested using another ELISA (Genedia HIV ½ ELISA 3.1, Green Cross Medical Science Corporation, Chungbuk, South Korea). Samples positive for both the ELISA were considered HIV sero-positive according to the two-test strategy for sentinel surveillance as per the NACO guidelines [22]. For quality assurance, all the HIV positive and 5% of HIV negative samples were retested at designated National HIV Reference Laboratory and the results of the retesting corroborated with the primary test results and thus confirmed them [19].

### Data Analysis

To understand the distribution of socio-behavioral characteristics and estimate the burden of HIV among participants, a descriptive analysis was conducted. Age, years of stay in current place of residence and days since last sex with a man were analyzed as continuous variables whereas age group (<25 years/25–34 years/≥35 years), literacy status (illiterate/up to 5^th^ standard/6^th^ to 10^th^ standard/11^th^ to Graduation), occupation (unemployed/student/labors, servants, workers/transport workers/hotel staff/small business/service), reason for coming to service point (collect condoms/for official work/drop-in-center, meet other MSM/recreational purpose), self-identified category of MSM (Double-decker/Panthi/Kothi), having sex with a female in last six months (no/yes), receiving/paying money for having sex with a man in last one month (no/yes) and injecting drug abuse in last one year (no/yes) were dealt with as categorical variables. The distributions of the characteristics across the strata of HIV sero-status were also determined and compared using unpaired t-test (for continuous variables), Chi-square test (categorical variables with enough cell size) and Fisher’s exact test (categorical variables with small cell size). Simple logistic regression analysis was performed to estimate individual associations between socio-behavioral characteristics and HIV sero-status. Finally, multiple logistic regression method was used to determine associations between each socio-behavioral factor and HIV sero-positivity while adjusting for all others using Adjusted Model 1 and adjusting for those for which bivariate results were significant using Adjusted Model 2. All analyses were done using SAS version 9.3.

## Results

Mean age of the participants was 28.3 years. A substantial proportion was illiterate (46.1%) and unemployed (27.2%). The population seemed to be mostly stable. About 36.2% of the subjects visited the site for official work. Majority identified themselves as Kothi (57%). Mean duration since last sex with a man was about 2 days, 85.2% had no recent heterosexual activity, 80.3% didn’t exchange money for having sex with men and 13.6% were HIV positive (Table 1).

**Table 1:** Distribution of socio-demographic, behavioral factors and HIV sero-status among MSM attending HIV sentinel site in Nagaland during 2010–11 (N^1^ = 243).

Continuous variable		Mean	Variance
Age (in years)		28.30	42.08
Years of stay in current place of residence		18.15	75.05
Days, since last sex with a man (missing = 1)		1.87	5.99
Categorical variable	Categories	Frequency	%
Age group	Very young (< 25 years)	71	29.22
	Young (25–34 years)	125	51.44
	Older (≥35years)	47	19.34
Literacy status	Illiterate	112	46.09
	Up to 5th standard (Primary school)	23	9.47
	6th to 10th standard (Middle school)	69	28.40
	11th to Graduation (High school & above)	39	16.05
Occupation	Unemployed	66	27.16
	Student	6	2.47
	Laborers/Servants/Workers	63	25.93
	Transport workers	6	2.47
	Hotel staff	5	2.06
	Small business	86	35.39
	Service	11	4.53
Reason for coming to the service point	Collect condoms	73	30.04
	For official work	88	36.21
	Drop in center/Meet other MSM	40	16.46
	Recreational purpose	42	17.28
Self-identified type (missing = 1)	Double decker	84	34.71
	Panthi	20	8.26
	Kothi	138	57.02
Having sex with female in last 6 months	No	207	85.19
	Yes	36	14.81
Received or paid money for having sex with a man in last 12 months	No	195	80.25
	Yes, paid money	4	1.65
	Yes, received money	35	14.40
	Both	9	3.70
IV drug use in last 1 year	No	243	100.00
	Yes	0	0.00
HIV test result	Negative	210	86.42
	Positive	33	13.58

^1^N, Total number of participants.

Distribution of age, duration of stay in current place of residence, reason for visiting the site and heterosexual activity were found to be similar while those of literacy, self-identified categories and duration since last sex with a man varied significantly across the strata of HIV sero-status (Table 2). Among the HIV positive participants, mean age was approximately 30 years, 66.7% belonged to the 25–34 years age group, 36.4% were educated up to middle-school level, 33.3% were unemployed, 33.3% visited the site for official purposes, majority were Kothis (78.8%), only 12.1% reported heterosexual activity in last six months while 15.2% admitted to have both paid and received money while having sex with a man in last month (Table 2).

**Table 2:** Distribution^1^ of socio-demographic and behavioral characteristics^2^ across HIV sero-status among MSM attending HIV sentinel site in Nagaland during 2010–11 (N^3^ = 243).

Socio-demographic and behavioral characteristics		HIV -ve			HIV +ve			p value
Continuous variable		n^4^	Mean	Var^5^	n^4^	Mean	Var^5^	
Age (in years)		210	28.04	43.63	33	29.94	30.00	0.078
Years of stay in current place of residence		210	18.45	77.16	33	16.19	59.05	0.130
**Days, since last sex with a man** (missing = 1)		209	1.99	6.62	33	1.09	1.40	0.001
Categorical variable	Categories	n^4^	%		n^4^	%		p value
Age group	Very young (< 25 years)	67	31.90		4	12.12		0.061
	Young (25–34 years)	103	49.05		22	66.67		
	Older (≥ 35years)	40	19.05		7	21.21		
**Literacy status**	Illiterate	105	50.00		7	21.21		0.004
	Up to 5th standard (Primary school)	20	9.52		3	9.09		
	6th to 10th standard (Middle school)	57	27.14		12	36.36		
	11th to Graduation (High school & above)	28	13.33		11	33.33		
Occupation	Unemployed	55	26.19		11	33.33		-
	Student	6	2.86		0	0.00		
	Laborer/Servants/Workers	60	28.57		3	9.09		
	Transport workers	6	2.86		0	0.00		
	Hotel staff	2	0.95		3	9.09		
	Small business	75	35.71		11	33.33		
	Service	6	2.86		5	15.15		
Reason for coming to the service point	Collect condoms	63	30.00		10	30.30		0.233
	For official work	77	36.67		11	33.33		
	Drop-in-center/Meet other MSM	31	14.76		9	27.27		
	Recreational purpose	39	18.57		3	9.09		
**Self-identified type** (missing = 1)	Double decker	79	37.80		5	15.15		0.022
	Panthi	18	8.61		2	6.06		
	Kothi	112	53.59		26	78.79		
Having sex with female in last 6 months	No	178	84.76		29	87.88		0.795
	Yes	32	15.24		4	12.12		
Received or paid money for having sex with a man in last 12 months	No	170	80.95		25	75.76		-
	Yes, paid money	4	1.90		0	0.00		
	Yes, received money	32	15.24		3	9.09		
	Both	4	1.90		5	15.15		

^1^ Boldface indicates characteristic with significantly different distribution (p<0.05) across the strata of HIV sero-status.

^2^ “-”refers to situations where valid p value could not be determined due to absence of adequate number of observation in individual categories

^3^ N: Total number of participants

^4^ n: Number of participants in each subcategories

^5^ Var: Variance.

With reference to MSM aged <25 years, those aged 25–34 years (Unadjusted Odds Ratio: OR 3.58; 95% Confidence Interval: 95% CI 1.18–10.84 and Adjusted Model 1Odds Ratio: AOR 3.89; 95% CI 1.02–14.80) and 35 yrs or more (OR 2.93; 95% CI 0.81–10.64 and AOR 2.17; 95% CI 0.42–11.33) had higher odds of being HIV positive in both adjusted and unadjusted model although for the older age group results were not statistically significant (Table 3). Compared to the illiterate, middle (OR 3.16; 95% CI 1.18–8.47 and AOR 3.44; 95% CI 1.02–11.62) and high-school/college (OR 5.89; 95% CI 2.09–16.59 and AOR 4.47; 95% CI 1.12–17.83) level educated MSM were much more likely to be HIV positive among the participants in both bivariate and multivariate analyses. In the unadjusted model, laborers/servants/workers were less likely (OR 0.25; 95% CI 0.07–0.94) while hotel staff (OR 7.50; 95% CI 1.12–50.28) and service-holders (OR 4.17; 95% CI 1.08–16.10) were much more likely to be sero-positive for HIV compared to their unemployed counterparts. Kothis had much higher odds of having HIV with reference to double-deckers in both unadjusted and adjusted models (OR 3.67; 95% CI 1.35–9.96 and AOR 3.60; 95% CI 1.08–11.99). Frequency of sexual act with a man seemed to be negatively associated with risk of HIV although the adjusted analysis lacked power (OR 0.71; 95% CI 0.52–0.97 and AOR 0.67; 95% CI 0.43–1.04). Participating MSM who did receive and pay money for sex with a man during last one year were much more likely to be HIV infected than those who didn’t (OR 8.50; 95% CI 2.14–33.79 and AOR 7.32; 95% CI 1.27–42.27). In the second multivariate model (Adjusted Model 2) after adjustment for only those which were significant in the bivariate analyses, results did not change much.

**Table 3:** Association^1^ of socio-demographic and behavioral characteristics^2^ with HIV sero-positivity among MSM attending HIV sentinel site in Nagaland, 2010–11 (N^3^ = 243).

Categorical variable	Categories	Unadjusted			Adjusted Model 1^4^			Adjusted Model 2^5^		
		OR^6^	95% CI^7^	p value	OR^6^	95% CI^7^	p value	OR^6^	95% CI^7^	p value
Age group	Very young (< 25 years)	Reference			Reference			Reference		
	Young (25–34 years)	**3.58**	**1.18,10.84**	**0.024**	**3.89**	**1.02,14.80**	**0.046**	3.53	0.96,12.88	0.057
	Older (≥35years)	2.93	0.81,10.64	0.102	2.17	0.42,11.33	0.357	2.05	0.43,9.67	0.365
Literacy status	Illiterate	Reference			Reference			Reference		
	Up to 5th standard (Primary school)	2.25	0.54,9.44	0.268	3.14	0.61,16.08	0.169	2.91	0.59,14.40	0.190
	6th to 10th standard (Middle school)	**3.16**	**1.18,8.47**	**0.022**	**3.44**	**1.02,11.62**	**0.046**	**3.32**	**1.04,10.60**	**0.043**
	11th to Graduation (High school & above)	**5.89**	**2.09,16.59**	**0.001**	**4.47**	**1.12,17.83**	**0.034**	**4.47**	**1.23,16.28**	**0.023**
Occupation	Unemployed	Reference			Reference			Reference		
	Student	-			-			-		
	Laborer/Servants/Workers	**0.25**	**0.07.0.94**	**0.041**	0.36	0.08,1.57	0.173	0.36	0.08,1.58	0.176
	Transport workers	-			-			-		
	Hotel staff	**7.50**	**1.12,50.28**	**0.038**	2.14	0.19,23.71	0.535	2.51	0.27,23.50	0.421
	Small business	0.73	0.30,1.81	0.502	0.58	0.20,1.70	0.316	0.57	0.19,1.66	0.300
	Service	**4.17**	**1.08,16.10**	**0.039**	2.22	0.39,12.55	0.369	2.02	0.38,10.89	0.412
Years of stay in current place of residence		0.97	0.93,1.01	0.164	1.00	0.94,1.06	0.870	NA^8^		
Reason for coming to the service point	Collect condoms	Reference			Reference			NA^8^		
	For official work	0.90	0.36,2.26	0.822	0.69	0.22,2.20	0.530			
	Drop in center/Meet other MSM	1.83	0.67,4.96	0.236	0.65	0.18,2.35	0.512			
	Recreational purpose	0.49	0.13,1.87	0.293	0.82	0.17,4.01	0.801			
Self-identified type (missing = 1)	Double decker	Reference			Reference			Reference		
	Panthi	1.76	0.32,9.78	0.521	2.48	0.29,21.52	0.596	2.24	0.34,14.86	0.405
	Kothi	**3.67**	**1.35,9.96**	**0.011**	**3.60**	**1.08,11.99**	**0.026**	**3.68**	**1.16,11.67**	**0.027**
Days, since last sex with a man (missing = 1)		**0.71**	**0.52,0.97**	**0.034**	0.67	0.43,1.04	0.072	0.68	0.45,1.02	0.059
Having sex with female in last 6 months	No	Reference			Reference			NA^8^		
	Yes	0.77	0.25,2.33	0.640	0.83	0.15,4.41	0.822			
Received or paid money for having sex with a man in last 12 months	No	Reference			Reference			Reference		
	Yes, paid money	-			-			-		
	Yes, received money	0.64	0.18,2.24	0.482	0.67	0.15,2.95	0.596	0.72	0.17,3.09	0.660
	Both	**8.50**	**2.14,33.79**	**0.002**	**7.32**	**1.27,42.27**	**0.026**	**6.65**	**1.24,35.64**	**0.027**

^1^ Boldfaced values indicate association with HIV sero-positivity for which p value < 0.05 (our assumed α level)

^2^ “-”refers to situations where valid OR could not be determined due to absence of observation in any of the subcategories

^3^ N: Total number of participants

^4^ Adjusted Model 1: Multivariate logistic regression model including all variables in the model

^5^ Adjusted Model 2: Multivariate logistic regression model including only those variables in the model for which p value was <0.05 in bivariate analysis

^6^ OR: Odds Ratio

^7^ CI: Confidence Interval

^8^ NA refers to those variables which were not included in the Adjusted Model 2.

## Discussions

HIV sero-positivity level among MSM varied considerably (0.36% to 14.98%) across Indian states [4,9,12,13,23,24] despite a consistently elevated risk for HIV acquisition compared to general population [25]. Social stigma and ill-defined sexual identity might well be responsible for these observed variations and the resultant possibility of underestimation of the true burden of HIV in this hard-to-reach population always remained a concern. During the solitary HSS conducted so far among MSM in the hilly north-eastern state of Nagaland, in 2011, HIV percentage positivity was 13.58%., which was 3, 18 and 50 times of the 2011 estimates for HIV prevalence among MSM in India, adult HIV prevalence of Nagaland and India respectively. This observed burden of HIV among MSM here was comparable or even higher than contemporary levels reported from similar settings in Brazil (ranged between 5.2%-23.7%) [26], China (12.4%) [27], Thailand (1.9%) [28] and South Africa (13.2%) [29] but lower than those reported from Baltimore (37.5%) [30] and Peru (22.3%) [31]. Keeping the current slowly declining trend of HIV among MSM in this country in mind, this alarmingly high HIV burden among MSM in Nagaland, might be considered as one of the greatest public health concern.

The mean age of the participating MSM was 28.3 years, which was either similar [6,12,28] or a bit higher [9,24,32] than most of the contemporary observations from other parts of India and abroad while in China the observed value (36 years) was even higher [27]. Alike other Indian and international studies [24,27,29,33,34], relatively higher age was associated with increased odds of being HIV positive. With increasing age accumulating of risk due to widening of the network, increased number of partners, more diverse mixing patterns and more high-risk behaviors might be the potential explanations.

Corroborating with prior studies elsewhere in India, majority of the participant was illiterate [6,35]. The literacy level of the MSM in China was found to be comparatively higher (75% had middle school or higher level of education) [27].But contrary to the previous findings across the states of India [12,23,24,35,36] and in China [27], among participants of HSS, 2011 in Nagaland, higher education was associated with increased HIV positivity. Probably in the remote, hilly state of Nagaland, educated MSM had increased scope of getting engaged in high-risk behaviors. On the other hand it was also possible that among educated MSM those who perceived their high-risk, participated more than their apparently low-risk counterparts.

Similar to prior studies, proportions of unemployed, laborers/servants/workers and owners of small businesses were higher among the study subjects compared to other occupational groups [23,24]. Among these participants, laborers/servants/workers were found to have the lowest risk while hotel staffs and service-holders were at much higher risk of acquisition of HIV compared to the unemployed. While some of these observations corroborated with previous findings [35,37], it seemed that occupation with more economic facility and scopes to be secretly involved in diverse sexual relationships were associated with higher HIV risk.

Being identified as an MSM in India entails immense social stigma and discrimination compelling this population to become mostly hidden [6,33,38]. Among self-identified MSM subtypes in this country, Panthis are more hidden than Double-deckers and Kothis. Due to their feminine appearance, attire and behavior, Kothis were most easily identifiable. This explains why in most of the studies involving MSM in India, the majority of the participants were Kothis [33,35,38,39]. In the current study also the proportion of Panthis were much lower (8.26%) compared to other subgroups although participation of a comparatively good proportion (34.7%) of Double-deckers indicated that the initial HSS in Nagaland was able to recruit a diverse population of MSM in terms of self-identified subgroups. Due to their predominantly receptive role, the risk of acquisition of HIV among Kothis was likely to be higher than others [8,9] as also observed in this study. Multilevel risk-reduction strategies emphasizing de-stigmatization of MSM may help in better identification and coverage of MSM subtypes in most part of India including Nagaland.

The mean duration of “days since last sex with a man” was found to be less than 2 days indicating that the participants of the HSS, 2011 among MSM in Nagaland were sexually pretty much active and decreased frequency of sexual activity was associated with lower likelihood of HIV sero-positivity. Sexually more active MSM probably had more likely to get engaged in risky sexual behavior (including unprotected anal intercourse) leading to higher risk of HIV acquisition [40, 41].

Compared to prior studies, much lower (14.8%) proportion of recruited MSM reported heterosexual activities in last six months [6,9,24]. We didn’t find any association between being engaged in heterosexual activities and the HIV risk but this might well have been influenced by sparse data problem. As a result of not having an overriding distinct homosexual identity, under social pressure, many Indian MSM usually get engaged in heterosexual activities and act as a potential bridge population (in terms of spreading HIV) between high-risk MSM group and their regular female partners [6,9,13,17,23,32,42].

Relatively small proportion of subjects (19.8%) in the current study admitted to have exchanged money for having sex with a man in last one month. This behavior was reported to be much more common in this population and strongly associated with higher HIV acquisition risk by several investigators in India [9,13,28,35,43], Peru (33.5% in clinic-based and 21.2% in community based samples) [31] and Thailand (approximately 50%) [28].Among the MSM who took part in HSS, 2011 in Nagaland those who did pay and receive money for having sex with men in last month had much higher odds of being HIV positive compared to those who never exchanged money for such purpose.

Being an IDU had been found to be a relatively common [28] and well-established risk factor for HIV among MSM [13]. In the current study none of the participants admitted to be IDU which was a bit surprising keeping in the mind the fact that in Nagaland, HIV epidemic was initially concentrated among IDUs. Further research is needed to determine whether this finding was a result of misreporting due to lack of social desirability or use of injecting drugs has nowadays become so uncommon among MSM in this part of India.

The study population comprised of voluntary participants from the MSM living in Nagaland. Thus the results might suffer from potential lack of generalizability. Any extrapolation of the findings to infer about MSM of Nagaland as a whole should be done with caution. Selection bias was a possibility if the participation of the subjects somehow got affected by their HIV status and socio-behavioral characteristics. As the MSM attending the sentinel site were recruited irrespective of their characteristics or HIV sero-status, the possibility of selection bias was probably less. The study was cross-sectional in nature hence causal interpretations of the observed associations are not recommended. Sparse data problem was also an issue in some of the analyses, especially multivariate, leading to insufficient statistical power for some of the observations. Sensitive behavioral information (heterosexual activity, days since last sex with man, exchanging money for sex with a man, being IDU) were all self-reported in nature and thus vulnerable to recall and social desirability bias. The social desirability component was probably minimized due to the anonymous data collection method. To make the interview process less time-consuming, the questionnaire had to be short. Hence information on several covariates and some more behavioral factors could not be collected.

## Conclusions

Burden of HIV seemed to be alarmingly high among MSM in Nagaland. Middle and older age, middle school or higher education, occupations like hotel staffs and service-holders, being Kothi, increased frequency of sex with a man, having received and paid money for sex with a man were strongly associated with higher HIV risk. Despite of all potential limitations, this initial insight into the burden of HIV and its correlates in this yet unexplored hard-to-reach population of MSM might be considered as a valuable baseline document for Nagaland. Intervention program targeting the high risk MSM population might help in controlling HIV in this high prevalent state in India.
